# Haplotype frequencies of 17 Y-chromosomal short tandem repeat loci from the Cukurova region of Turkey

**DOI:** 10.3325/cmj.2011.52.703

**Published:** 2011-12

**Authors:** Ayse Serin, Husniye Canan, Behnan Alper, Yasar Sertdemir

**Affiliations:** 1 Cukurova University Medical Faculty, Department of Forensic Medicine, Adana, Turkey; 2 Cukurova University Medical Faculty, Department of Biostatistics, Adana, Turkey

## Abstract

**Aim:**

To investigate the distribution of 17 Y-short tandem repeat (STR) loci in the population of the Cukurova region of Turkey.

**Methods:**

In the period between 2009 and 2010, we investigated the distribution of 17 Y-STRs in a sample of 249 unrelated healthy men from the Cukurova region of Turkey. Genomic DNA was extracted with InstaGene matrix and Y-STRs were determined using the AmpFISTR Yfiler PCR amplification kit. Gene and haplotype diversity values were estimated using the Arlequin software. To compare our data to other populations, population pairwise genetic distances and associated probability values were calculated using the Y Chromosome Haplotype Reference Database Web site software.

**Results:**

At 17 Y-STR loci we detected 148 alleles. The lowest gene diversity in this region was 0.51 for DYS391 and the highest 0.95 for DYS385a/b. Haplotype diversity was 0.9997 ± 0.0004. We compared our data with haplotype data of other Turkish populations and no significant differences were found, except with Ankara population (Φ_st_ = 0.025, *P* = 0.018). Comparisons were also made with the neighboring populations using analysis of molecular variance of the Y-STR loci genetic structure and our population was nearest to Lenkoran-Azerbaijani (Φ_st_ = 0.012, *P* = 0.068) and Iranian Ahvaz population (Φ_st_ = 0.007, *P* = 0.173), followed by Greek (Φ_st_ = 0.026, *P* = 0.000) and Russian (Φ_st_ = 0.048, *P* = 0.000) population. Other countries like Portugal, Spain, Italy, Egypt, Israel (Palestinian Authority Area), and Taiwan showed a high genetic distance from our population.

**Conclusion:**

Our study showed that Y-STR polymorphisms were a powerful discrimination tool for routine forensic applications and could be used in genealogical investigations.

Human Y chromosome short tandem repeats (Y-STR) are tandemly repeated regions of 2-7 base pair units in the non-recombining region of human Y chromosome. Since human Y-STR markers are inherited without recombination, they are transmitted from father to son unchanged. This makes the study of Y chromosome polymorphisms very useful, especially in population histories, forensic applications, and paternity analysis (1,2).

The haplotype frequency of Y-STRs is important in the calculation of match probability in forensic cases. It is also important in anthropology and phylogenetic studies (1-5). Y chromosome haplotypes from global populations are stored in Y Chromosome Haplotype Reference Database (YHRD) (*www.yhrd.org*). The International Society of Forensic Genetics recommended the use of regional Y-STR haplotype databases to verify that no population sub-structure exists before pooling data from different regions (3). Since there is a lack of Y-STR data on Turkish population in eastern Mediterranean area, we analyzed 249 unrelated Turkish men using 17 Y-STR markers including DYS19, DYS389I, DYS389II, DYS390, DYS391, DYS392, DYS393, DYS385a/b, DYS438, DYS439, DYS437, DYS448, DYS456, DYS458, DYS635, and Y-GATA H4 from the Cukurova region in the eastern Mediterranean region of Turkey.

## Participants and methods

Blood samples were collected from 249 unrelated healthy and voluntary donors from the Cukurova region of Turkey. We obtained the samples from the Balcali Hospital staff and from the casework in our forensic laboratory. Genomic DNA was extracted from 6-µL whole blood samples by using recommended protocol with InstaGene matrix (BioRad, Alfred Nobel Dr, Hercules, CA, USA) or Qiagen DNA micro kit (Valencia, CA, USA). Polymerase chain reaction amplification was performed following manufacturer’s instructions (Applied Biosystems, Foster City, CA, USA). The loci DYS19, DYS389I, DYS389II, DYS390, DYS391, DYS392, DYS393, DYS385a/b, DYS438, DYS439, DYS437, DYS448, DYS456, DYS458, DYS635, and Y-GATA H4 were analyzed using the AmpFiSTR Y-filer^TM^ kit (Applied Biosystems). These amplification reactions were performed using a GeneAmp PCR System 9700 (Applied Biosystems). The ABI 3130 Genetic Analyzer (Applied Biosystems) was used for genetic typing. Allele designations were based on the comparison with the allelic ladders included in the AmpFISTR Y-filer^TM^ kit (Applied Biosystems), using the Gene Mapper software, version 3.2.

Blind testing and evaluation was performed according to the quality assessment scheme provided by the YHRD. After a successful certification, the data in this study from Turkish populations were submitted to YHRD, and YA003668 accession number was received. The study was approved by the Ethics Committee of the University of Cukurova, Faculty of Medicine.

### Statistical analysis

Gene and haplotype diversity values were estimated using the Arlequin software (6,7). In order to examine the relationship of the studied population and the neighboring populations, population pair-wise genetic distances (Φ_st_) and associated probability values (*P* values) were calculated using analysis of molecular variance (AMOVA), with an online YHRD tool (8). Pair-wise genetic distance is an analogue of the commonly used F_st_ that measures the evolutionary distance between individual haplotypes, defined as Φ_st_ = (πt-πs)/πt, where πt is the nucleotide sequence diversity across the entire set of populations and πs is the average nucleotide sequence diversity within populations (9,10). To test for significance, *P* values were calculated (10 000 permutations). The DYS385 marker was not included in the population comparisons, because two alleles were not assigned to the individual locus (ie, DYS385a or DYS385b). Significance level was set at *P* < 0.05.

## Results

We detected 148 alleles at the 17 Y-STR loci in 249 samples. Using the present set of Y-STR markers, 240 different haplotypes were identified, 232 of which were unique and 7 were found in 14 individuals (each was found in 2 individuals) and 1 was found in 3 individuals. The observed haplotype diversity was 0.9997 ± 0.0004.

The most polymorphic marker was DYS385a/b with a genetic diversity (GD) value of 0.95 and the least polymorphic was DYS391 with a GD value of 0.51. Apart from DYS385, the loci DYS458, DYS635, and DYS389II showed the highest GD values. Different loci had a different number of alleles, with the most recorded alleles at DYS458.

A number of intermediate alleles was seen at DYS458 – 12.2, 16.2, 17.2, 18.2, 19.2, and 20.2. The frequency of intermediate alleles at DYS458 was 16.8% of the samples. Only one duplicate allele (allele 15 and allele 16) was observed at DYS19 locus. One null allele and one intermediate allele (17.2) were observed at DYS448 (Table 1).

**Table 1:** Allele frequencies and gene diversities of the 17 Y- short tandem repeat loci in 249 samples from Turkish men

Allele	DYS19	DYS389I	DYS389II	DYS390	DYS391	DYS392	DYS393	DYS438	DYS439	DYS437	DYS448	DYS456	DYS458	DYS635	GATAH4	Alleles	DYS385
9					0.060			0.273	0.004						0.020	9,11	0.004
10					0.634	0.028		0.434	0.112						0.056	9,15	0.004
11		0.004			0.297	0.635	0.020	0.145	0.418						0.450	9,17	0.004
12	0.004	0.189			0.008	0.040	0.522	0.145	0.357						0.325	10,14	0.004
12,2													0.004			10,19	0.004
13	0.096	0.582				0.201	0.329	0.004	0.104			0.016	0.020		0.141	11,12	0.004
14	0.522	0.221				0.072	0.092		0.004	0.530		0.144	0.028		0.008	11,13	0.010
15	0.233	0.004				0.016	0.032			0.329		0.538	0.205			11,14	0.149
15,16	0.004															11,15	0.044
16	0.104					0.004	0.004			0.137		0.213	0.233			11,19	0.008
16,2													0.004			11,20	0.004
17	0.036					0.004				0.004	0.004	0.080	0.185			11,22	0.004
17,2											0.004		0.036			12,12	0.016
18											0.040	0.008	0.129			12,13	0.032
18,2													0.068			12,14	0.012
19											0.357		0.036	0.012		12,15	0.040
19,2													0.024			12,16	0.032
20											0.382		0.004	0.104		12,17	0.032
20,2													0.020			12,18	0.020
21				0.036							0.169		0.004	0.305		12,19	0.008
22				0.157							0.028			0.205		12,2	0.004
23				0.422							0.008			0.277		13,13	0.016
24				0.261							0.004			0.060		13,14	0.064
null											0.004					13,15	0.036
25				0.116										0.012		13,16	0.084
26				0.036										0.012		13,17	0.036
27			0.028											0.012		13,18	0.064
28			0.080													13,19	0.024
29			0.357													13,20	0.012
30			0.341													14,14	0.016
31			0.157													14,15	0.032
32			0.032													14,16	0.016
33			0.004													14,17	0.012
																14,18	0.008
																14,19	0.004
																14,20	0.004
																15,15	0.012
																15,16	0.020
																15,17	0.024
																15,18	0.004
																16,16	0.016
																16,17	0.024
																16,18	0.004
																16,19	0.004
																	
Gene diversity	0.65	0.58	0.73	0.72	0.51	0.55	0.61	0.70	0.68	0.59	0.70	0.64	0.85	0.78	0.67		0.95

Due to the limited number of markers reported in other publicly available Turkish population databases, the comparative analysis was performed with a minimal European Y-STR haplotype comprising 7 loci – DYS19, DYS389I, DYS389II, DYS390, DYS391, DYS392, DYS393. Three groups of Turkish men: from Germany, Bulgaria, and Ankara (Turkey) were available for comparison. When these data were compared using AMOVA with each other and with our results from the Cukurova region, there was no significant difference among the results from Bulgaria, Germany, and the Cukurova region. When they were compared to the results from the Ankara region, there was a significant difference between Ankara population and the groups from Bulgaria (Φ_st_ = 0.033, *P* = 0.025) and the Cukurova region (Φ_st_ = 0.025, *P* = 0.018) (Table 2) (11-13).

**Table 2:** Analysis of molecular variance pairwise distance based on Φst values between the Turkish population samples*

Population	Ankara, Turkey	Bulgaria (Turks)	Germany (Turks)	Cukurova, Turkey
Ankara, Turkey	-	0.028^†^	0.139	0.018^†^
Bulgaria (Turk)	0.033	-	0.541	0.469
Germany (Turks)	0.009	-0.002	-	0.460
Cukurova, Turkey	0.025	-0.001	-0.000	-

*P values are shown above and Φ_st_ values below the diagonal.

†The level of significance is *P* < 0.05.

Our data were compared with the data for Russia, Egypt, Portugal, Israel (Palestinian Authority Area), Italy, Spain, Iran (Lenkoran-Azerbaijani-North Talysh), Greece, and Taiwan, for which the same set of Y-STR loci was available (8,14-21). Among the neighboring countries, Turkish population samples were nearer to Lenkoran-Azerbaijani (Φ_st_ = 0.012, *P* = 0.068) and Iranian-Ahvaz (Φ_st_ = 0.007, *P* = 0.173) than Greek (Φ_st_ = 0.026, *P* = 0.000) and Russian (Φ_st_ = 0.048, *P* = 0.000) population samples. A high genetic distance was observed between our population and other geographically more distant countries like Portugal, Spain, Italy, Egypt, Israel (Palestinian Authority Area), and Taiwan. Egypt (Φ_st_ = 0.055, *P* = 0.034) was closer than Spain (Φ_st_ = 0.074, *P* = 0.000), Portugal (Φ_st_ = 0.069, *P* = 0.000), Italy (Φ_st_ = 0.095, *P* = 0.000), Israel (Φ_st_ = 0.089, *P* = 0.000), and Taiwan (Φ_st_ = 0.375, *P* = 0.00) (Table 3).

**Table 3:** Analysis of molecular variance pairwise distance based on Φst values between Cukurova (Turkey) and 10 other populations*^†^

	Population samples										
	Ahvaz, Iran	Alpujarra de la Sierra, Spain	Archangelsk, Russian Federation	Aswan, Egypt	Central Portugal, Portugal	Palestinian Authority Area, Israel,	Lenkoran, Azerbaijan	Modena, Italy	Northern Greece, Greece	Taiwan	Cukurova, Turkey (Turkish)
Ahvaz, Iran	-	0.000	0.000	0.049	0.000	0.000	0.104	0.000	0.011	0.000	0.173
Alpujarra de la Sierra, Spain	0.139	-	0.000	0.011	0.527	0.000	0.000	0.433	0.000	0.000	0.000
Archangelsk, Russian Federation	0.080	0.111	-	0.001	0.000	0.000	0.000	0.000	0.000	0.000	0.000
Aswan, Egypt	0.049	0.112	0.173	-	0.009	0.018	0.174	0.005	0.006	0.000	0.034
Central Portugal, Portugal	0.130	-0.002	0.123	0.094	-	0.000	0.000	0.016	0.000	0.000	0.000
Israel, Palestinian Authority Area	0.049	0.168	0.170	0.074	0.166	-	0.000	0.000	0.000	0.000	0.000
Lenkoran, Azerbaijan	0.017	0.099	0.125	0.027	0.081	0.090	-	0.000	0.000	0.000	0.068
Modena, Italy	0.178	-0.001	0.142	0.132	0.009	0.217	0.114	-	0.000	0.000	0.000
Northern Greece, Greece	0.024	0.137	0.086	0.079	0.132	0.127	0.066	0.159	-	0.000	0.000
Taiwan	0.422	0.433	0.479	0.436	0.403	0.469	0.359	0.429	0.427	-	0.000
Cukurova, Turkey (Turkish)	0.007	0.074	0.048	0.055	0.069	0.089	0.012	0.095	0.026	0.375	-

*P values are shown above and Φ_st_ values below the diagonal.

†The level of significance is *P* < 0.05.

## Discussion

Our analysis of genetic polymorphisms of 17 Y-STR loci in the Cukurova population showed a high degree of haplotype diversity in the Cukurova population. It also found DYS385 to be one of the most informative markers.

In the last fifteen years, genetic structure of Y chromosome has been determined in several countries (14-24). A few studies including 7, 9, or 11 Y-STRs were also conducted in Turkey (13,25-27). However, our study presents the first population data for 17 Y-STR loci in eastern Mediterranean region of Turkey.

It seems more complex to construct a Y-STR database than that of unlinked autosomal markers, since the whole haplotype must be a type for each sample. The practical value of Y-STR databases will be greatly increased with typing of each individual at as many loci as possible, as opposed to typing a great number of individuals at a small number of loci. In order to increase the discrimination power of Y chromosome haplotypes, an increased number of Y-STRs is used. Our results are important since we examined a large number of loci and had a big sample size. Such information is of crucial importance for genetic epidemiology and population genetic purposes (28,29).

The most striking locus in our population data was DYS458, at which a number of intermediate alleles were observed. Intermediate alleles of DYS458 were most frequently found in Northern and the Eastern Africa and the Caucasus (24,30,31). They have been reported to be less common in Europe (8). One intermediate allele named 17.2 was also observed at DYS448. YHRD search confirms that this allele has been reported in only 2 individuals. Such partial repeat variants occur at low frequencies but may be useful for better understanding of diversity within the Y chromosome gene pool (30). This is considered particularly useful for evaluating regional Y-chromosome variation or migrations occurring in the recent past. Moreover, it increases the discrimination power of DNA evidence. Therefore, forensic community needs to share information on the occurrence of these variants.

We compared our results with 3 Turkish population groups (Bulgaria, Germany, and Ankara) (11-13). We could not make a full comparison because these populations had available data on fewer loci than our population. Therefore, we compared only minimal European Y-STR haplotype and found significant differences only when Ankara group was compared to samples from Bulgaria and the Cukurova region (8).

We also compared it to the neighboring populations by using 15 loci haplotypes. The greatest similarities were found with the Lenkoran-Azerbaijani and Iranian-Ahvaz population, followed by Russian and Greek population. This similarity may be a result of historical and demographic reasons. Lack of similarity with other neighbors may be a result of genetic differences and/or a small number of studied individuals. Significant differences were observed from nearly all populations from the Far East.

This study does not reflect the genetic structure of whole Turkey, which could be considered a limitation. Furthermore, the comparison with the other populations was based on the samples available in the YHRD, which were occasionally rather small and thus perhaps not representative of their entire populations. Further studies are needed to determine the genetic structure of all Turkish populations, so that these data can be used for forensic and genealogical investigation and to gain understanding of some of the major demographic and historical events in this region. However, the analysis of Y-STR polymorphisms in our population using Yfiler provides a powerful discrimination tool for routine forensic applications and may serve in genealogical investigations.
